# CT Perfusion in Patients with Lung Cancer: Squamous Cell Carcinoma and Adenocarcinoma Show a Different Blood Flow

**DOI:** 10.1155/2018/6942131

**Published:** 2018-09-03

**Authors:** Alessandro Bevilacqua, Giampaolo Gavelli, Serena Baiocco, Domenico Barone

**Affiliations:** ^1^DISI (Department of Computer Science and Engineering), University of Bologna, Viale Risorgimento 2, Bologna 40136, Italy; ^2^ARCES (Advanced Research Center on Electronic Systems), University of Bologna, Via Toffano 2/2, Bologna 40125, Italy; ^3^Istituto Scientifico Romagnolo per lo Studio e la Cura dei Tumori (IRST) IRCCS, Meldola 47014, Italy; ^4^DEI (Department of Electrical, Electronic and Information Engineering), University of Bologna, Viale Risorgimento 2, Bologna 40136, Italy

## Abstract

**Objectives:**

To characterize tumour baseline blood flow (BF) in two lung cancer subtypes, adenocarcinoma (AC) and squamous cell carcinoma (SCC), also investigating those “borderline” cases whose perfusion value is closer to the group mean of the other histotype.

**Materials and Methods:**

26 patients (age range 36-81 years) with primary Non-Small Cell Lung Cancer (NSCLC), subdivided into 19 AC and 7 SCC, were enrolled in this study and underwent a CT perfusion, at diagnosis. BF values were computed according to the maximum-slope method and unreliable values (*e.g.,* arising from artefacts or vessels) were automatically removed. The one-tail Welch's t-test (*p*-value <0.05) was employed for statistical assessment.

**Results:**

At diagnosis, mean BF values (in [mL/min/100g]) of AC group [(83.5 ± 29.4)] are significantly greater than those of SCC subtype [(57.0 ± 27.2)] (*p*-value = 0.02). However, two central SCCs undergoing artefacts from vena cava and pulmonary artery have an artificially increased mean BF.

**Conclusions:**

The different hemodynamic behaviour of AC and SCC should be considered as a biomarker supporting treatment planning to select the patients, mainly with AC, that would most benefit from antiangiogenic therapies. The significance of results was achieved by automatically detecting and excluding artefactual BF values.

## 1. Introduction

Tumorigenesis involves angiogenesis, a complex mechanism consisting in the generation of a vascular network nourishing the tumour that is highly disorganized [1]. Studying abnormal vascular patterns, and their temporal changes, may be essential for tissue characterization [2]. To this purpose, the interest in CT perfusion (CTp) methodologies has been recently confirmed [3], since CTp supplies both high spatial and temporal resolution and allows computing perfusion parameters from the analysis of the time concentration curves (TCCs) [4], generated by the contrast agent reaching the tumour. Among the most effective perfusion parameters allowing angiogenesis evaluation [5], the blood flow (BF) arises [6] mainly because of its high correlation with the tissue biomarker microvessel density (MVD) [7]. BF can be measured by considering only the first passage of the contrast medium, this requiring a short-time examination permitting to minimize the radiation dose given to the patient [8, 9]. Clinical applications of BF information computed at diagnosis include the lesion characterization [10], especially relevant for inoperable patients, who need to schedule non-surgical treatments [11]. Or else, higher baseline BF values in patients with advanced lung carcinoma could suggest a better response to therapy [12].

The differences in BF values between responders and nonresponders have prompted a tumour hemodynamic characterization, which also embodies cancer histological subtypes. Characterizing the perfusion of non-small cell lung cancer (NSCLC) has shown to provide useful information concerning their status, in particular as regards their hypoxia degree, deeply affecting tumour response to treatment [13]. In particular, it was shown that the adenocarcinoma (AC) subtype has a significantly lower hypoxia degree than the squamous cell carcinoma (SCC) one [14]. Various perfusion parameters have been reported to differ between lung cancer subtypes [13, 15], showing discordant results [16–21]. However, when considering these discordant findings, we must bear in mind that BF computation of lung tumours is exposed to several sources of error [22], arising from respiratory motion [23], CTp artefacts [24], and, not to forget, tumour location [25], which can affect the reliability of BF values [26], thus dimming the real nature of tumours and hampering development and standardization of biomarkers. As regards tumour location, its influence on BF values is rarely considered in CTp studies. Nonetheless, it has been shown that perfusion in central carcinomas is significantly lower than in peripheral ones, due to the different recruitment of vessels [27]. Nevertheless, also anatomical structures inside lesion, such as vessels and bronchi, can be responsible for jeopardizing perfusion analyses [26].

The aim of this study was to evaluate the characteristics of lung tumour at diagnosis (i.e., at baseline condition), newly investigating possible significant differences in perfusion between AC and SCC, the two predominant NSCLC phenotypes. Nevertheless, as previously shown, the literature presents discordant results in AC and SCC perfusion characterization, caused by too a high measurement variability, stemming from clinical and physiological factors as well as external causes (e.g., patient movements and artefacts). To reduce such a variability, we automatically detected and removed unreliable perfusion values. In addition, we examined lesions' position, central or peripheral, and their proximity to large vessels, studying whether and how this external factor could artificially affect histotype perfusion. Finally, for each histotype we also analyse the less representative lesions, whose perfusion values are shifted to the group mean value characterizing the other histotype.

## 2. Materials and Methods

*Study Population*. This study was approved by the Institutional Review Board (IRB) that waived informed consent for the retrospective data analysis of the patients. In addition, a written consensus was obtained by all patients before each study. Between September 2010 and April 2013, a total of 32 consecutive patients (22 men, 10 women; age range 36-81 years) with primary NSCLC, subdivided in 24 AC and 8 SCC, were identified and enrolled for data analysis. Tumour stage was determined in all patients according to the TNM classification of malignant tumours (seventh edition): 1 patient was diagnosed with stage IB, 3 patients with stage IIIA, 5 patients with stage IIIB, and 23 patients with stage IV.

The patients included in this study were selected according to the following criteria: over eighteen years old, with histologically verified NSCLC, and no prior history of chemotherapy, surgery, or thoracic radiation therapy. Patients were excluded if the longest axial lesion diameter was less than 10 mm in at least one slice (n=2), if the examination was severely affected by physics-based artefacts (n=4), in case of a clinically significant cardiovascular disease and a known history of deep vein thrombus or pulmonary embolus. The final population included 26 patients, 19 AC, and 7 SCC. Besides subtypes and staging, Table 1 includes lesion's location and position as the cancer features. For the sake of completeness, we also report the widest axial tumour section.

*CTp Protocol*. At diagnosis, the patients underwent axial CT perfusion (CTp) performed on a 256-slice CT system (Brilliance iCT, Philips Medical System, Best, The Netherlands), feet first in the supine position. Initially, an unenhanced low-dose full-body CT scan was performed to identify the target lesion at baseline conditions. Then, a 50 mL intravenous bolus of contrast agent (Iomeron, Bracco, Milan, Italy) was injected at 5 mL/s for axial cine contrast-enhanced CT, followed by a flush with physiological saline solution (30 mL), 5 mL/s). Five seconds later, each patient underwent a single acquisition of duration 25 seconds under breath-hold condition. This protocol yields 20 scans, each corresponding to different sampling instants, with 55 mm of z-coverage (11 slices ×  5-mm slice thickness, 0.4-sec rotation time, at 80 kV, 250 mA, 100 mAs). Image data are reconstructed to 220 cine images (512 × 512 pixel, 11 slices, 350 mm ×  350 mm, 5-mm slice spacing, 1.25-sec temporal resolution).

*Perfusion Maps*. A region of interest (ROI) in the descending aorta was selected as the input function. A second ROI was manually drawn in consensus [27] by two experienced radiologists (D.B. and G.G., 25-year experience each) on a reference slice, where the tumour showed the widest area, similar to what was done in [20, 27, 28]. Then, for each lesion, the radiologists performed a 3D alignment according to the procedure described in [29]. In order to obtain the TCCs relative to each voxel, the values of the temporal sequence were fitted using the sigmoid-shape model, arising from the Hill Equation [30], and the random sampling method described in [31]. The curve fitting is achieved employing an in-house fitting algorithm based on the nonlinear, least squares, Levenberg-Marquardt minimization algorithm (lsqcurvefit, MATLAB^©^; MathWorks, Natick, MA, USA). After TCCs computation, BF values, expressed in mL/min/100 g, were computed for AC and SCC subtypes using the maximum-slope method [32, 33] by considering the first-pass phase only [34] and representing each voxel with a one-compartment model, including both the intravascular and the extravascular spaces [35]. This allowed performing short-time examinations with great benefits for patients, in terms of absorbed radiation dose and examination quality, since motion artefacts were reduced by the possibility for patients to hold their breath.

Nonetheless, in this paper, we propose two methodological approaches to improve the reliability of results, that is, removing potential artefacts from perfusion maps through an accurate data analysis and separately analysing the “borderline cases”.

*Data Analysis*. Unreliable BF values were excluded from the analysis and highlighted in the colour map with the pink colour. In particular, BF values strictly lower than 1 mL/min/100 g were automatically removed, being considered unlikely compliant with physiological values and rather ascribable to numerical errors. Similarly, BF values undergoing high TCC fitting errors due to the presence of structures, such as blood vessels and bronchi, or dynamic artefacts, were automatically detected as unreliable through the method presented in [26]. Finally, mean BF values were computed for each examination and considered to identify hemodynamic differences between the two histological NSCLC subtypes, AC and SCC.

**Figure 1:**
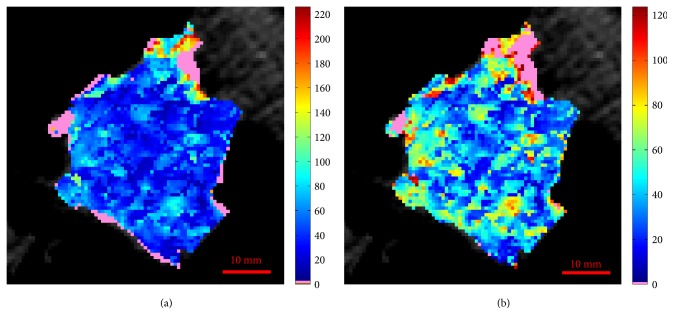
Perfusion maps of ID15 achieved through the use of the automatic denoising method (a) and by hand clipping the highest BF values (b). In pink, the unreliable values.

**Figure 2:**
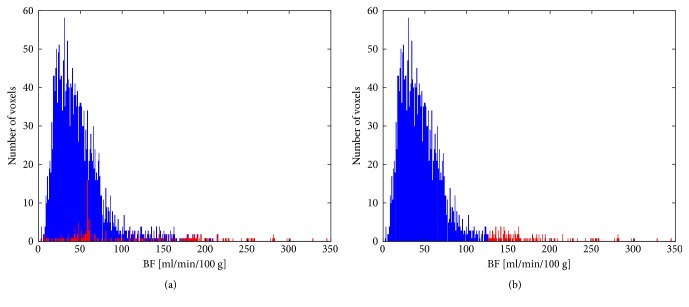
In blue, the histograms related to the original ID15 BF map and, in red, the BF values that were removed through the use of the automatic denoising method (a) and by hand clipping the highest BF values (b).

To better understand the implications of the denoising methods we used, in Figure 1 we report two BF maps related to ID15, obtained with the denoising method we used [26] (a) and by manually removing (i.e., clipping) the highest BF values (b), supposedly out of the physiological range, as it is normally done. The removed values are shown in the pink colour in both maps. As one can see, the denoising method removes unreliable regions, including the outer ones, that is, those suffering from partial volume effect, which pure clipping normally keeps. Nevertheless, this method preserves a range of BF values wider than clipping does. This behaviour is underlined by the BF histogram of Figure 2(b), showing that, by clipping, the highest BF values (in red) are removed independently from their reliability, and the range of values narrowed. On the other side, the BF histogram of Figure 2(a) highlights the clear advantage of the denoising method, which “saves” those high values which are generated with no appreciable errors. Furthermore, one can see how this method is able to even automatically remove unreliable low values falling within the physiological BF range. This explains why the range of BF values is wider, though the regions removed have a wider extent. Although these two maps are apparently very similar, the real benefit of the denoising method definitely arises in the subsequent analysis. For instance, as regards the perfusion analysis, the mean BF value of the clipped map (mean BF = 44.1 mL/min/100 g) is smaller than that of the corresponding denoised map (mean BF = 47.7 mL/min/100 g) by almost 10%. This underestimation of the mean BF value, due to the inclusion of unreliable BF values and exclusion of high BF values regardless of their reliability, could severely affect the statistical analysis of all perfusion studies. Of course, in the presence of bronchi, vessels, and, above all, artefacts this difference can be even larger.

*The “Borderline” Examinations*. After performing the automatic error analysis to detect and exclude unreliable values, we looked for the causes that could affect the perfusion of the “borderline” cases. Indeed, these are the most aspecific lesions of the two classes, whose parameter values are nearest to each other, which negatively affect the statistical significance of the study. If from one side they could simply originate from artefacts, from the other side, more interestingly, they can reflect the inherent variability of data and the intrinsic tumour properties.

### 2.1. Assessment of Results

The main purpose of this work is to determine whether AC and SCC are characterized by a different baseline perfusion. In order to assess the statistical significance of the differences in BF between AC and SCC subtypes, the one-tail Welch's t-test was used for mean, while the one-tail Wilcoxon Rank Sum test (also known as Mann-Whitney U-test) was used for median. For both, the statistical significance is achieved with p-value <0.05. Statistical analysis was performed using R software (version 3.0.1, The R Foundation for Statistical Computing).

As regards the analysis of the borderline cases, we examined several factors that may cause perfusion variability. We considered where tumours arose, right or left lung, since it is proved that they drain to different lymph node groups according to their position [36]. In this context, we also considered the tumour location, central or peripheral, since central tumours can almost completely fed by the bronchial circulation, while the peripheral tumours, mainly at their early growth stage, can have a significant pulmonary contribution [20]. Finally, we looked for the extrinsic effects on perfusion computation of beam hardening artefacts which, for instance, occur in tumour localized near bony regions of the chest and, also, where the contrast medium is highly concentrated [24].

#### 2.1.1. Tumour Location

For each examination, two radiologists examined the entire scan sequence so as to form in their mind the morphological structure of the lesion, also exploiting dynamic information. They divided tumours into three groups according to their locations, also reporting if they are in the left or right lung. A tumour was considered peripherally located if it is 20 mm of the costal pleura, within the pulmonary parenchyma without direct connection to mediastinal structures. A tumour is centrally located if it is 20 mm of the mediastinal structures, in a close relationship with the central airways. Otherwise, it is considered as an extended tumour (that could be either large or small). The two radiologists started by performing this analysis in double blind and, then, they reviewed together all cases to reach an agreement. This information is reported in Table 1.

## 3. Results

**Figure 3:**
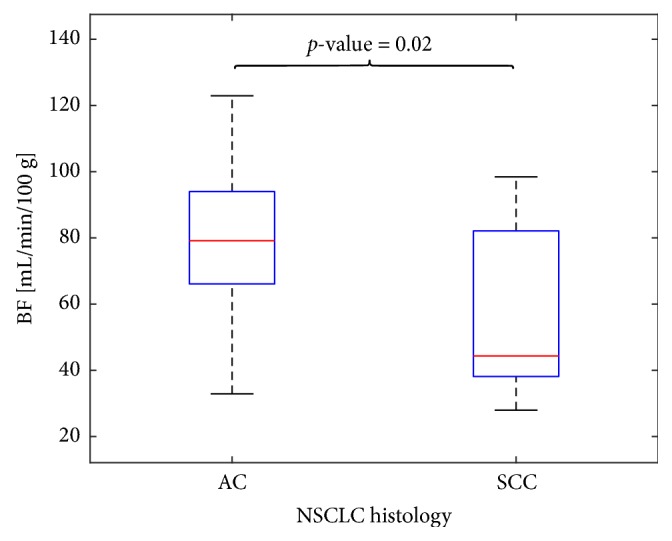
Box plots showing the BF values of AC (left) and SCC (right) lesions. The median is indicated as a red line in the boxes, whereas the vertical size gives the interquartile range.

**Figure 4:**
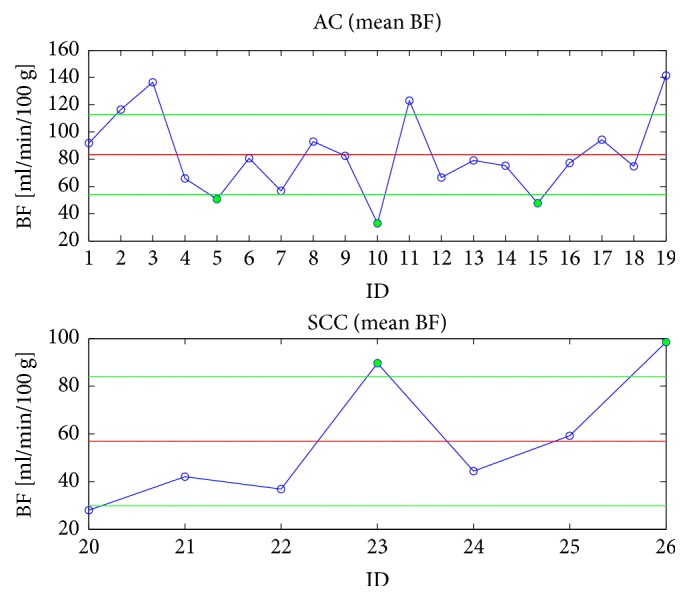
Mean BF values of AC (top) and SCC (bottom) examinations (blue circles), along with the corresponding group mean (solid red line) and SD (solid green line) values. The green circles highlight the examinations for each subtype that are closer to the mean value of the other subtype.

Table 2 resumes the main BF parameter values for AC and SCC subtypes, while Figure 3 graphically shows the distribution of BF values for the two subtypes. The outcome highlights that the baseline BF mean value of AC examinations is definitely greater than that of SCC ones (p-value = 0.02), as well as the BF median value (p-value = 0.03).  Figure 4 shows the averaged BF values of each AC (top) and SCC (bottom) examination. For reader's convenience, the samples have been joined and displayed with solid blue lines, whereas the group mean and standard deviation (SD) values are shown with solid red and green lines, respectively. As one can see, as it often happens each subtype may have some borderline examinations nearer to the group mean value of the other subtype (the green disks in Figure 4), which are responsible for reducing the statistical significance of the between-group mean difference. In particular, ID5 (mean BF = 50.5 mL/min/100 g), ID10 (mean BF = 32.9 mL/min/100 g), and ID15 (mean BF = 47.7 mL/min/100 g) are three AC examinations whose BF values are closer to the SCC group mean value, while ID23 (mean BF = 89.7 mL/min/100 g) and ID26 (mean BF = 98.5 mL/min/100 g) are two SCCs with mean values closer to AC group mean. In the next section, we consider other studies aiming at assessing perfusion values of AC and SCC, besides these borderline cases, which are analysed to assess whether their mean value really reflects phenotypical features.

## 4. Discussion

In the literature, studies concerning the perfusion characterization of AC and SCC report contrasting results. The authors in [16] indicate that AC is characterized by a more abundant blood supply than SCC, as the higher peak of their TCCs suggested. Moreover, also blood volume and flow-extraction product are significantly higher in AC than in SCC [14]. The authors in [17] also found that AC has apparently a higher perfusion than SCC, but these results were not statistically significant, although MVD is significantly more intense in AC than in SCC. Other studies [18–20] highlighted no differences in perfusion parameters among these two histological subtypes, also finding that they are characterized by a similar MVD [21].

**Figure 5:**
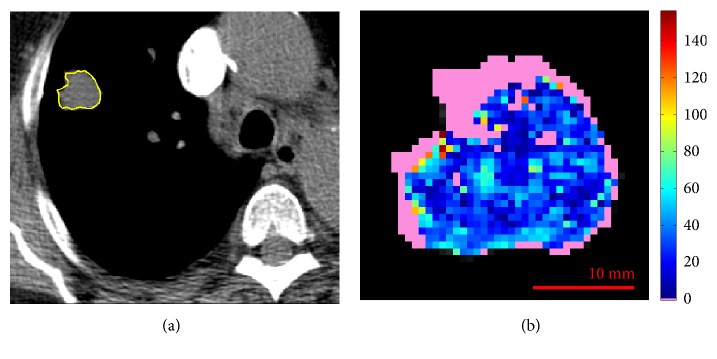
Reference slice (a) and perfusion map (b) related to ID10. In pink, the unreliable values.

**Figure 6:**
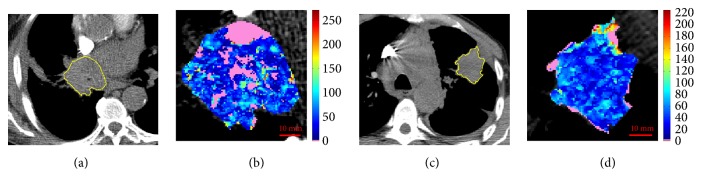
Reference slice and perfusion map related to ID5 (a, b) and ID15 (c, d). In pink, the unreliable values.

Among the possible causes for these discordant results, we propose to consider also those borderline cases whose perfusion values might be ascribed to motivation other than their phenotypical features. The first comment concerns ID10, the AC lesion characterized by the lowest perfusion and shown in Figure 5. This lesion is a very small peripheral carcinoma, one of the smallest examined, located in the subpleural parenchyma, probably characterized by a predominant pulmonary circulation, which could not have activated the angiogenesis process yet [37]. As regards the other two AC lesions with a low perfusion, ID5 in Figure 6(a) and ID15 in Figure 6(b), these share similar properties that could explain their low perfusion. In fact, both of them are large and extended lesions, presenting wide low-perfusion regions, maybe suggesting hypoxia, which lower the mean BF values. Altogether, these three cases seem not showing any external characteristic artificially altering their BF. For instance, ID19 (mean BF = 141.4 mL/min/100 g) is a central carcinoma, as large as ID15, with a high perfusion value (the highest one). As a matter of fact, lesions of such a dimension are often characterized by a hypoxic core, due to the presence of weak and disorganized capillaries characterizing tumour angiogenesis. These vessels, being more permeable than normal, increase the liquid of the extravascular space, causing the adjacent cells moving away from the vessels and, consequently, the low oxygenation of the surrounding tissue. However, the presence of segmental vessels inside ID19 still nourished the core of the lesion.

**Figure 7:**
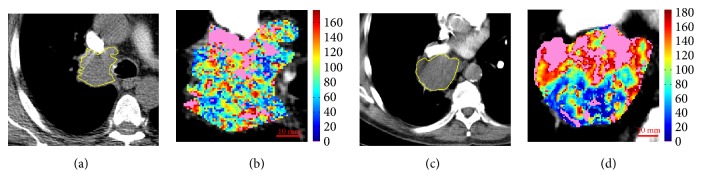
Reference slice and perfusion map related to ID23 (a, b) and ID26 (c, d). In pink, the unreliable values.

**Figure 8:**
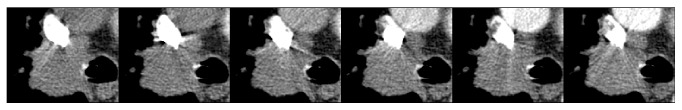
A sequence of six slices of ID23, referring to same couch position, shows the effect of beam hardening artefacts on lesions.

**Figure 9:**
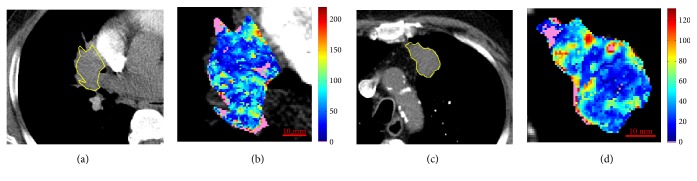
Reference slice and perfusion map related to ID25 (a, b) and ID21 (c, d). In pink, the unreliable values.

**Figure 10:**
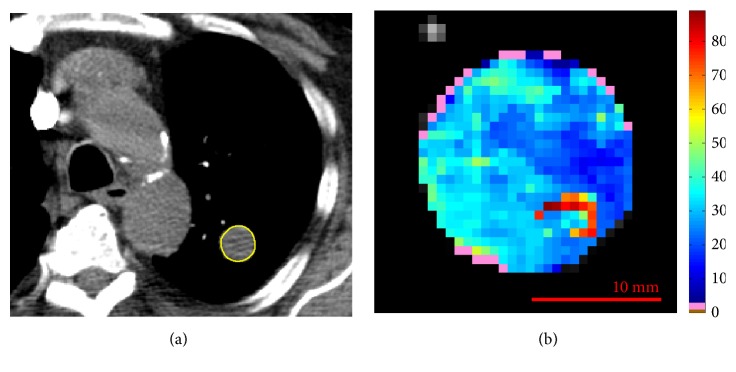
Reference slice (a) and perfusion map (b) related to ID20. In pink, the unreliable values.

As far as SCC are concerned, the two examinations ID23 and ID26 (Figure 7), showing a higher perfusion compared to the others SCC, share a common feature. Indeed, they are both central SCC lesions positioned at the right lung, directly connected to the vena cava and the pulmonary artery, respectively. This particular location, in proximity of these large blood vessels, may yield several artefacts during image acquisition, as shown in the original slices of Figure 7, which are responsible for an artificial increasing of BF values. A detail of six artefactual slices of ID23, referred to the same couch position, is shown in Figure 8. Nonetheless, one other central lesion, ID25 (mean BF = 59.3 mL/min/100 g) in Figure 9(a), suffers from moderate artefacts, while the last one, ID21 (mean BF = 42.0 mL/min/100 g) in Figure 9(b), is not artefactual. It is worth mentioning that if the artefacts in ID23, ID25, and ID26 were removed manually, BF values for SCCs would rise to mean BF = 63.5 mL/min/100 g and SD BF = 36.9 mL/min/100 g, this yielding the difference between the overall means of the histotypes not to be statistically significant (p-value = 0.08). As a marginal note, it is interesting to see how the SCC lesion characterized by the lowest mean BF value in our court, ID20 (mean BF = 28.0 mL/min/100 g), shown in Figure 10, is staged IB.

**Figure 11:**
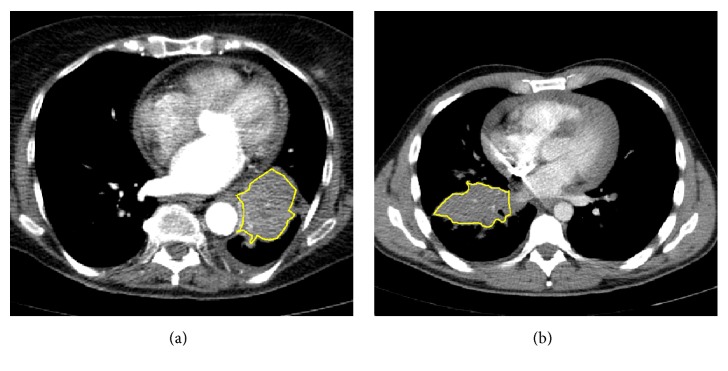
AC examinations ID2 (a) and ID11 (b) undergoing beam hardening artefacts.

At the end, we analysed the mean BF values of each lesion in relation to position and location. We report that each of the four peripheral ACs in the left lung has a mean value greater than the group mean, and the three right peripheral lesions have a lower mean BF, probably because of two bronchial arteries usually running to the left lung, while one only to the right lung. Similarly, all the extended AC lesions, neglecting their position, have a mean value lower than the group mean, except for ID2 and ID11 (Figures 11(a) and 11(b), respectively), that represent large lesions undergoing light beam hardening artefacts, from left and right atrium, respectively.

The present study newly investigated the perfusion baseline characteristics of AC and SCC, the two major NSCLC subtypes, after that the literature has reported discordant outcomes. We addressed the BF mean value, commonly used as significant statistical parameter in several studies [7, 38]. Our results show that, before treatments, the AC histological type has a BF mean value significantly greater than SCC subtype, which generally shows a lower perfusion associated with an increased presence of necrotic areas. These results arise from the reliability analysis of the BF maps and are enforced by the analysis of borderline cases.

The reliability assessment, carried out through an automatic and objective error detection method [26], allowed removing the anatomical structures (mainly blood vessels and bronchi) and regions undergoing artefacts that could compromise the correct interpretation of perfusion maps, thus considerably improving their reliability. In particular, automatically removing and excluding all the artefactual regions from the subsequent analysis is probably the main reason why this study found a clear statistical significance of the different BF properties of AC and SCC histotypes.

Besides improving perfusion reliability, we analytically examined the borderline cases, that is, those most aspecific examinations of the two histotypes, whose perfusion values are so near to the mean value of the other histotype as to partly lose their representativeness. In particular, we looked for some external causes, besides phenotypical properties, which could motivate the mean BF value of each borderline case. As far as the three ACs are concerned, we could not find any external cause that could explain their lower BF values. Rather, the wide range covered by the AC histotype could be suggestive of the existence of subpopulations with different perfusion behaviours. Although there is no statistical evidence regarding probable effects of tumour's position and location on the BF mean values, we found some interesting tendencies regarding subgroups of the AC histotypes that could support this hypothesis and are worth investigating separately in a dedicated study. On the contrary, the predominantly central position of SCCs makes them prone to beam hardening artefacts, due to high contrast medium concentration in large neighbouring vessels, such as vena cava and the pulmonary artery. Indeed, it is known that artefacts may yield false results in tumours localized near to large central vessels [27]. In particular, we proved that without automatically removing those artefactual regions from perfusion computation in two borderline SCCs, their BF mean values would expectedly be higher so as that the difference between AC and SCC means would not be significant any more. This could explain the discordant results emerging in the literature regarding the perfusion characterization of these lung cancer subtypes, since the “noise” emerging from the colour maps was almost disregarded. As an example, the study carried out in [15] reports group mean and SD BF values for AC (74.7 ± 28.2 mL/min/100 g) and SCC (68.7 ± 32.1 mL/min/100 g) that are so near to each other as to conclude that their difference is not statistically significant. However, a deep analysis of SCCs could highlight that they underwent artefacts and using our same method to remove them could have led to the opposite conclusion.

As an added value of this research, we want to stress how these results have been achieved using a short-time, dose-saving, protocol that could foster other studies aiming at investigating the peculiarities of AC subgroups, as far as BF is concerned. Nonetheless, increasing the examination time would jeopardize the possibility for patients to hold their breath, and motion artefacts introduced after a 25-sec period would worsen the quality of the image sequence [27].

This study has also some limitations. The first is the relatively small cohort of patients. However, other works in the literature reported a similar number of examinations, such as that in [38] (22 AC, 8 SCC), or smaller, like in [18] (14 AC, 9 SCC), [20] (18 AC, 5 SCC), and [14] (6 AC, 8 SCC). Nonetheless, removing the unreliable perfusion values improved the statistical significance of the examinations at our disposal. The second limitation stems from the first one, as the number of examinations prevented us from exploring BF properties of possible AC subgroups. Finally, we have studied the BF only. However, considering other parameters was beyond the purpose of this research.

## 5. Conclusions

The main purpose of this work was to investigate the BF properties of AC and SCC at diagnosis, before treatment, and clear perfusion differences emerged from these NSCLC cancer subtypes that should be considered during their treatment planning. Nonetheless, we introduced two methodological contributions that allowed this study, with a non-large size cohort and a short-time protocol, to achieve a clear outcome. These contributions, which could benefit other cancer perfusion studies, consist in the use of a method to improve the reliability of single examinations and the accurate analysis of those borderline lesions less characterizing for the histotype they belong to. In particular, we believe it is important to explicitly investigate the causes that may be responsible for those values because, besides permitting to detect values that are artificially high (or low), the borderline lesions could contain even more valuable information than the other ones.

Among the practical advantages, the capability of achieving more accurate results could prevent the need of using a higher tube voltage, for instance, when investigating central carcinomas, which reduces the sensitivity to the contrast medium and increases the exposure of the patient [27].

We encourage the authors of all the previous studies on AC and SCC to review their analysis in the light of the methodological approaches presented in this research. In this age of personalised medicine, a non-invasive profiling of the tumour in terms of perfusion characteristics, apparently an independent surrogate biomarker, could have important implications in treatment strategy, particularly in the identification of the patients, mainly with AC, that will most benefit from antiangiogenic therapies.

## Figures and Tables

**Table 1:** Summary of the histological diagnosis, tumour stage, position, location, and area of the widest tumour slice relative to each lesion.

Patient ID	Diagnosis	Stage	Position	Location	Size [*cm*^2^]
ID1	AC	IV	Left	Peripheral	2.4
ID2	AC	IV	Left	Extended	15.9
ID3	AC	IV	Left	Peripheral	4.6
ID4	AC	IV	Right	Extended	7.0
ID5	AC	IV	Right	Extended	16.1
ID6	AC	IV	Right	Peripheral	15.0
ID7	AC	IIIA	Right	Extended	5.9
ID8	AC	IV	Left	Peripheral	1.5
ID9	AC	IV	Right	Extended	29.0
ID10	AC	IIIA	Right	Peripheral	2.5
ID11	AC	IV	Right	Extended	20.3
ID12	AC	IV	Right	Extended	3.9
ID13	AC	IV	Right	Peripheral	0.6
ID14	AC	IV	Left	Extended	2.5
ID15	AC	IV	Left	Extended	10.8
ID16	AC	IV	Right	Extended	1.9
ID17	AC	IV	Left	Peripheral	1.5
ID18	AC	IV	Left	Central	3.8
ID19	AC	IIIB	Right	Central	10.7
ID20	SCC	IB	Left	Peripheral	2.1
ID21	SCC	IV	Left	Central	7.2
ID22	SCC	IV	Right	Peripheral	5.2
ID23	SCC	IIIB	Right	Central	10.3
ID24	SCC	IIIB	Right	Extended	22.8
ID25	SCC	IV	Right	Central	8.2
ID26	SCC	IIIB	Right	Central	16.1

**Table 2:** BF stratified by NSCLC subtypes.

NSCLC Subtypes	BF [mL/min/100 g]				
	Mean	Median	Minimum	Maximum	SD
AC	83.5	79.2	33.0	141.3	29.4
SCC	57.0	44.3	28.0	98.4	27.2

Note: SD = standard deviation.

## Data Availability

The data used to support the findings of this study are included within the article.
